# HER3 and downstream pathways are involved in colonization of brain metastases from breast cancer

**DOI:** 10.1186/bcr2603

**Published:** 2010-07-06

**Authors:** Leonard Da Silva, Peter T Simpson, Chanel E Smart, Sibylle Cocciardi, Nic Waddell, Annette Lane, Brian J Morrison, Ana Cristina Vargas, Sue Healey, Jonathan Beesley, Pria Pakkiri, Suzanne Parry, Nyoman Kurniawan, Lynne Reid, Patricia Keith, Paulo Faria, Emilio Pereira, Alena Skalova, Michael Bilous, Rosemary L Balleine, Hongdo Do, Alexander Dobrovic, Stephen Fox, Marcello Franco, Brent Reynolds, Kum Kum Khanna, Margaret Cummings, Georgia Chenevix-Trench, Sunil R Lakhani

**Affiliations:** 1 Molecular & Cellular Pathology, The University of Queensland Centre for Clinical Research, & School of Medicine, Building 918/B71, RBWH complex, Brisbane, 4029, Australia; 2 Cancer Genetics and Molecular Pathology, The Queensland Institute of Medical Research, 300 Herston Road, Brisbane, 4006, Australia; 3 Departamento de Anatomia Patológica, Universidade Federal de São Paulo, EPM, 754 Rua Napoleão de Barros, São Paulo, 04024-000, Brazil; 4 Biomolecular and Biomedical Science, Griffith University, 170 Kessels Road, Brisbane, 4011, Australia; 5 Centre for Magnetic Resonance, The University of Queensland, St Lucia, Brisbane, 4072, Australia; 6 Lembaga Eijkman, Eijkman Institute, Diponegoro 69, Jakarta, 10430, Indonesia; 7 Departamento de Patologia, Instituto Nacional de Câncer, 23 Praça Cruz Vermelha, Rio de Janeiro, 20230-130, Brazil; 8 Departamento de Patologia, Laboratório Salomão & Zoppi, 48 Rua Correia Dias, São Paulo, 04104-000, Brazil; 9 Department of Pathology, Medical Faculty of Charles University in Plzen, Husova 3, 306 05, Czech Republic; 10 Sydney West Area Health Service, Institute of Clinical Pathology and Medical Research, University of Sydney, Darcy Road, Sydney, 2145, Australia; 11 Translational Oncology, Sydney West Area Health Service, Westmead Millennium Institute, University of Sydney, Darcy Road, Sydney, 2145, Australia; 12 Department of Pathology, Peter MacCallum Cancer Centre, St Andrews Pl, East Melbourne, 3002, Australia; 13 Queensland Brain Institute, The University of Queensland, St Lucia, Brisbane, 4072, Australia; 14 Signal Transduction, The Queensland Institute of Medical Research, 300 Herston Road, Brisbane, 4006, Australia; 15 Pathology Queensland: The Royal Brisbane & Women's Hospital, Herston Road, Brisbane, 4029, Australia; 16 Current address - University of Florida, McKnight Brain Institute,100 S. Newell Drive, Gainesville, 32611, USA

## Abstract

**Introduction:**

Metastases to the brain from breast cancer have a high mortality, and basal-like breast cancers have a propensity for brain metastases. However, the mechanisms that allow cells to colonize the brain are unclear.

**Methods:**

We used morphology, immunohistochemistry, gene expression and somatic mutation profiling to analyze 39 matched pairs of primary breast cancers and brain metastases, 22 unmatched brain metastases of breast cancer, 11 non-breast brain metastases and 6 autopsy cases of patients with breast cancer metastases to multiple sites, including the brain.

**Results:**

Most brain metastases were triple negative and *basal-like*. The brain metastases over-expressed one or more members of the HER family and in particular HER3 was significantly over-expressed relative to matched primary tumors. Brain metastases from breast and other primary sites, and metastases to multiple organs in the autopsied cases, also contained somatic mutations in *EGFR, HRAS, KRAS*, *NRAS *or *PIK3CA*. This paralleled the frequent activation of AKT and MAPK pathways. In particular, activation of the MAPK pathway was increased in the brain metastases compared to the primary tumors.

**Conclusions:**

Deregulated HER family receptors, particularly HER3, and their downstream pathways are implicated in colonization of brain metastasis. The need for HER family receptors to dimerize for activation suggests that tumors may be susceptible to combinations of anti-HER family inhibitors, and may even be effective in the absence of *HER2 *amplification (that is, in triple negative/basal cancers). However, the presence of activating mutations in *PIK3CA*, *HRAS, KRAS *and *NRAS *suggests the necessity for also specifically targeting downstream molecules.

## Introduction

Among women with breast cancer, 30% to 40% will develop metastatic disease. The natural history of metastatic breast cancer to the brain is of symptomatic disease in 10% to 20% of these patients and a dismal mean survival of six months following diagnosis [1,2]. Associations with younger age, p53 positivity, estrogen receptor (ER) negative and epidermal growth factor receptor 1 (EGFR) and two (HER2) positive cancers have been reported [3-5]. The epidermal growth factor receptor family comprises four receptors, HER1 to 4. Upon activation, hetero or homo-dimerization occurs, followed by phosphorylation of specific tyrosine residues in the intracellular domain, stimulating signaling cascades mediated mainly by AKT and MAPK and the regulation of cell proliferation, angiogenesis, migration and survival [6,7].

Basal-like tumors are generally high grade, negative for ER, progesterone receptors (PgR) and HER2 (that is, *triple negative*) [8]. The current dogma would predict that these tumors are unlikely to respond to endocrine and trastuzumab-based therapy and no targeted therapy is currently available, although clinical trials are ongoing [8]. Despite being node negative, a proportion of patients subsequently present with distant metastases, particularly to the brain [9,10]

Using autopsy records of breast cancer patients, Paget [11] demonstrated a non-random pattern of metastatic spread. This suggested that tumor cells (the *seed*) could have a specific affinity for the microenvironment of certain organs (the *soil*). In agreement, animal models demonstrate that particular sets of genes can increase the potential of breast cancer cell lines to colonize specific distant sites, for example, bone, lung [12,13]; and brain [14,15].

The cancer *mutatome *is very complex, with more than 140 CAN genes identified which are mutated at a significant frequency in cancer [16,17]. The genomic landscape of breast cancer is also very complex and heterogeneous, with different subgroups of tumours (luminal, basal, HER2) harboring different types and patterns of mutations [18]. There is also evidence that breast cancer cell lines with a basal phenotype have a higher frequency of mutations in *BRAF, KRAS*, and *HRAS *than luminal breast cancer cell lines [19-21].

We have analyzed a relatively large and rare set of human tumors to elucidate the mechanisms involved in colonization of the brain. Samples studied involved matched pairs of primary breast cancer and brain metastases, unmatched brain metastases, non-breast brain metastases and autopsy cases of breast cancer patients with metastases to multiple sites, including the brain. We provide evidence of increased activation of HER3 and downstream pathway molecules in brain metastases from breast cancer and suggest that the inhibition of HER family receptors, even in the absence of *HER2 *gene amplification (for example, triple negative/basal cancers), could play a significant role in the management of patients with brain metastases from breast cancer. In addition, we demonstrated the possible fallacies of this approach without considering the presence of somatic activating mutations in downstream molecules [22-24].

## Materials and methods

Additional detailed methodologies (see Additional file 1). The study was approved by the local research ethics committees under the project number UQ2005000785 and RBHW 2005/22.

### Clinical samples

All human clinical samples studied were available as formalin fixed-paraffin embedded (FFPE) tumor blocks. Cohorts collected were: i) 39 matched pairs of primary breast cancer and brain metastases; ii) 22 unmatched brain metastases from breast cancer; iii) 11 brain metastases from non-breast sites (one melanoma, one colorectal, six lung, one prostate and two renal cell carcinomas); and iv) 26 tumor samples (primary breast cancer and metastases to multiple sites, including brain) from six autopsy cases of patients who died of metastatic breast cancer (the primary breast cancer from one case was not available). The tumors were reviewed by three pathologists (LDS, MC and SRL) and analyzed by immunohistochemistry and chromogenic *in situ *hybridization (CISH) on tissue microarrays. Immunohistochemistry for EGFR, HER2, HER3, HER4, CD44 and CD24 was also done on whole sections.

### Gene expression analysis

RNA was extracted from FFPE samples and the expression of 512 cancer related genes was analyzed using the DASL assay (cDNA-mediated annealing, selection extension and ligation, Illumina Inc., San Diego, California, USA) [25]. All data and protocols for DASL analysis can be found at the Gene Expression Omnibus repository (Accession number GSE14690) (see also additional file 1). Real-time PCR using TaqMan^® ^gene expression assays (Applied Biosystems, Inc, Carlsbad, California, USA ) and immunohistochemistry were performed to validate the expression of specific genes.

### Somatic mutation analysis

Twelve matched pairs of primary breast tumors and corresponding brain metastases, nine non-breast brain metastases and 26 tumor samples from the six autopsy cases were subjected to primer extension and MALDI-TOF mass spectrometry using the OncoCarta^® ^Panel Assay v1.0 (Sequenom Inc., San Diego, California, USA) of 238 mutations in 19 oncogenes [26]. All mutations in samples for which there was sufficient DNA remaining were validated by High Resolution Melt (HRM) [27] analysis, iPLEX (using newly designed PCR and extension primers that differed from the OncoCarta primers), repeat OncoCarta analysis, and/or direct sequencing if the Mutant Allele Proportion (MAP) was >30% (Table 1 and Additional file 2, Table S2). In addition, we were able to validate the *EGFR *E746_A750del mutation in four cases with a mutation-specific antibody [28].

**Table 1:** Somatic mutations identified by OncoCarta and ER, PgR and HER family of receptors assessment

Matched breast primary-brain metastasis pairs												
**Case ID#**	**Site**	**ER - PgR- HER 1-2-3-4**	***EGFR***		***NRAS***		***PIK3CA***					

			**Mutation**	**MAP**	**Mutation**	**MAP**	**Mutation**	**MAP**				

**1**	**brain**	ER-, PgR-, HER1-, HER2+, HER3-, HER4-										

	**breast**	ER-, PgR-, HER1-, HER2+, HER3-, HER4-										

**2**	**brain**	ER-, PgR-, HER1-, HER2-, HER3+, HER4-			Q61R^O, H, I, S^	39.50%						

	**breast**	ER-, PgR-, HER1-, HER2-, HER3+, HER4-			Q61R^O, H, I, S^	38.30%						

**4**	**brain**	ER-, PgR+, HER1+, HER2-, HER3-, HER4-										

	**breast**	ER-, PgR+, HER1+, HER2-, HER3-, HER4-										

**6**	**brain**	ER-, PgR+, HER1+, HER2-, HER3-, HER4-										

	**breast**	ER-, PgR+, HER1+, HER2-, HER3-, HER4-										

**7**	**brain**	ER-, PgR-, HER1+, HER2-, HER3-, HER4-			Q61R^I, S^	34.4%						

	**breast**	ER-, PgR-, HER1+, HER2-, HER3-, HER4-			Q61R^I, S^	34.1%						

**8**	**brain**	ER-, PgR-, HER1-, HER2+, HER3+, HER4-										

	**breast**	ER-, PgR-, HER1-, HER2+, HER3-, HER4-										

**9**	**brain**	ER-, PgR-, HER1-, HER2+, HER3-, HER4-					H1047R^S^	79.50%				

	**breast**	ER-, PgR-, HER1-, HER2+, HER3-, HER4-					H1047R^S^	79.50%				

**10**	**brain**	ER-, PgR-, HER1+, HER2-, HER3+, HER4-					E545K^H, NVP^	23.40%				

	**breast**	ER-, PgR-, HER1+, HER2-, HER3-, HER4-					E545K^H, NVP^	18.20%				

**11**	**brain**	ER-, PgR-, HER1-, HER2+, HER3-, HER4+										

	**breast**	ER-, PgR-, HER1-, HER2+, HER3-, HER4+										

**12**	**brain**	ER-, PgR+, HER1-, HER2-, HER3+, HER4-										

	**breast**	ER-, PgR+, HER1-, HER2-, HER3+, HER4-										

**13**	**brain**	ER-, PgR-, HER1+, HER2-, HER3-, HER4-										

	**breast**	ER-, PgR-, HER1+, HER2-, HER3-, HER4-	N771_P772>SVDNR	12.10%								

**14**	**brain**	ER-, PgR-, HER1+, HER2-, HER3-, HER4-										

	**breast**	ER-, PgR-, HER1+, HER2-, HER3-, HER4-										

**Unmatched brain metastases from primary lung, colon, melanoma and kidney tumours**												

**Case ID#**	**Site**	**ER - PgR- HER 1-2-3-4**	***EGFR***		***HRAS***		***KRAS***		***NRAS***		***PIK3CA***	

			**Mutation**	**MAP**	**Mutation**	**MAP**	**Mutation**	**MAP**	**Mutation**	**MAP**	**Mutation**	**MAP**

**D2**	**melanoma**	n.a.									E545K^H, I, Y^	30.80%

**D3**	**colon**	n.a.					G12C^S^	38.90%				

**D4**	**lung**	n.a.	E746_A750del^A^	21.00%								

**D5**	**lung**	n.a.										

**D6**	**lung**	n.a.	E746_A750del^A, NVI^	14.40%	G13S^**I**^	17.30%			G12C^I;S^	35.70%		

**D7**	**lung**	n.a.							G12C^**O;I**^	9.70%		

**D8**	**lung**	n.a.					G12C^S^	39.90%				

**D9**	**lung**	n.a.									E545K^H^	13.30%

**D10**	**kidney**	n.a.	E746_A750del^A, I^	9.10%					G12C^**I, S**^	35.20%		

A, validated by immunohistochemistry using a mutation-specific antibody; ER, estrogen receptor; H, validated by High Resolution Melt analysis; I, validated by iPLEX; ID, case number identification; MAP, Mutant Allele Proportion estimated by OncoCarta; NVI, not validated by iPLEX; NVP, no further validation possible because no DNA remained; O, validated by repeat OncoCarta; PgR, progesterone receptor; S, validated by sequencing; Y, sequencing did not work for this sample; n.a., not accessed.

## Results

### Clinical and pathological features

The median age at diagnosis was 48.5 years and the median time for the development of brain metastasis was 3.5 years. All but one of the series of primary breast cancers and all brain metastases were grade 3 invasive ductal carcinomas-no specific type (IDC-NST) [29]. The remaining tumor pair was a grade 2 mucinous carcinoma. The autopsy samples comprised four grade 3 and one grade 2 IDC-NST.

### ER, PgR, HER2, 'Basal' markers and stem cell markers (non-autopsy cases)

**Figure 1:**
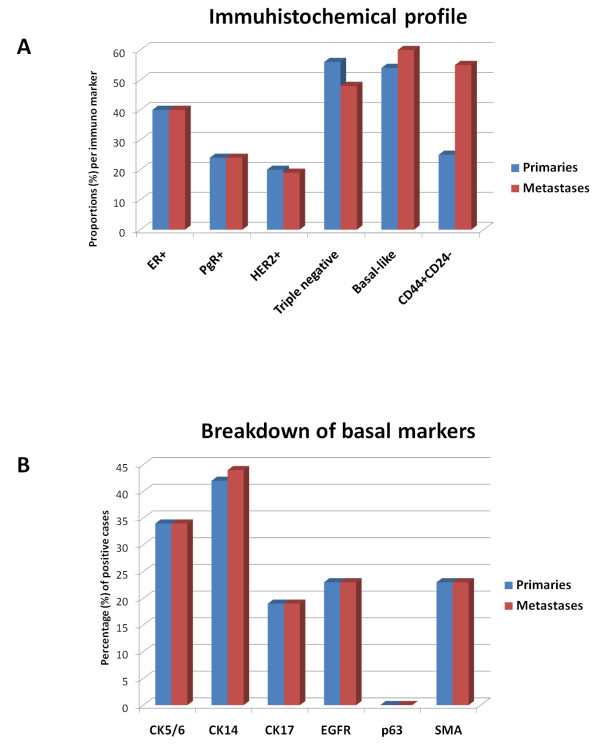
**Immunohistochemical profile of primary breast and brain metastases**. **A **- Immunohistochemical analysis of matched primary breast and brain metastases. The graph depicts percentages of positive cases in each category. ER and PR were considered positive when >10% cells showed staining, HER2 was considered positive when IHC showed 3+ staining (>30% positive cells) or CISH showed gene amplification. Triple negative tumors were negative for ER, PR and HER2. CD44+/CD24- immunohistochemistry was assessed on serial sections and positivity was expression in >10% cells. **B **- Breakdown of basal markers. A tumor was regarded as *basal *if any of the following markers were positive (CK5/6, CK14, CK17, p63, SMA, and EGFR) with >10% cells showed staining.

Immunohistochemistry data are summarized in Figure 1A, B (see also additional file 2, Table S1 and Figure S1). It was noteworthy that 60% and 76% of the tumors were negative for ER and PR, respectively, with complete concordance between primary and metastases. Seventy-seven percent (77%) and 81% of the unmatched brain metastases were also ER and PR negative, respectively. Twenty percent (20%) and 19% of the primary breast tumors and metastases, respectively, had correlated over-expression of HER2 (3+ staining) and all of these showed gene amplification using CISH. Twenty percent (20%) of the unmatched metastases were also HER2+. Fifty-six percent (56%) of the primary tumors and 48% of the matched metastases were triple negative and of these, 60% were positive for at least one of the basal markers respectively (CK14, CK5/6, CK17, EGFR and SMA). Overall, 54% of the primary and 60% of the metastases were of *basal *phenotype (irrespective of ER, PR and HER2 status), confirming enrichment in this cohort over the normal distribution in breast cancer [8]. Noteworthy, EGFR staining was seen mainly in the periphery of the tumor where there was contact with non-neoplastic brain parenchyma [30]. A higher proportion of brain metastases had a putative stem cell-like phenotype (CD44^+^/CD24^-^) compared to the primaries, 55% versus 25%, (Figure 1A). Fifty-one percent (51%) of the primary tumors had a Ki-67 index higher than 10% in contrast to matched and unmatched metastases that had 86% and 85% of samples with index higher than 10%.

### Gene expression profiling

**Figure 2:**
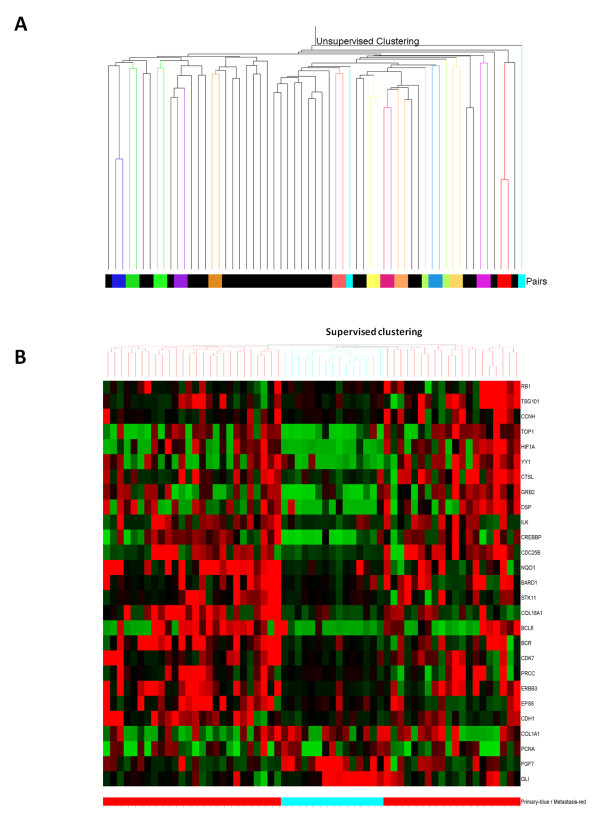
**Gene expression profiling of brain metastases**. **A **- Unsupervised hierarchical clustering of DASL gene expression data from 22 unmatched (black color bar) and 15 matched primary and brain metastases (other colors bars). Thirteen out of 15 matched samples are clustering together. **B **- Heatmap and dendogram showing clustering of the samples based on the 27 genes differentially expressed between primary tumors (blue line bar) and brain metastases (red line bar).

The availability of good quality RNA and stringent filtering of the DASL data yielded gene expression profiling data on 37/61 brain metastases from breast cancer (15/39 from matched pairs and 22/22 from unmatched metastases) and 15 matched primaries. Unsupervised analysis highlighted a strong similarity between primary tumors and their matched metastases (Figure 2A). Only 20 genes were differentially expressed between the matched primaries and metastases. This may be a consequence of the overall strong similarity between primaries and metastases [31] coupled with the sample size (n = 30) and number of genes analyzed (n = 512 cancer genes in the DASL panel) [32]. Comparison between primaries and all metastases (matched and unmatched) identified 27 statistically significant, differentially expressed genes (Figure 2B). Supplementary Figure 2 (see Additional file 2, Figure S2) depicts principal component analysis showing good separation of the primaries and metastases using this 27-gene list. All 20 genes identified in the matched pair analysis were part of this 27-gene set. Among this 20-gene set, were *HER3 *and one of its downstream target molecules *GRB2 *[33], hypoxia related molecule *HIF1-alfa*, MAPKinase cascade related protein *CREBBP*, cell cycle regulator RB1 and proliferation related genes *CCNH, CDK7 *and *CDC25B*. Since the brain is rich in neuregulin 1 [34,35] and this is a ligand for HER3, we hypothesized that the neuregulin-HER3 activation was important in allowing breast cancer cells to colonize the brain.

### HER family receptors and downstream molecules expression

*HER3, EGFR, HER2, HER4 *and *HIF1-alfa *expression was assessed using quantitative RT-PCR (see Additional file 2, Figure S3) in 12 matched breast/brain samples for which DASL data and RNA were available. Similar to the DASL data, 10 cases showed increased fold change by RT-PCR of *HER3 *gene expression relative to their matched primaries ranging from 1.12 to 5.8 and with an average of 2.4. Immunohistochemistry for HER3 was similar, showing positivity in 11/37 (29.7%) of the primary tumors, 22/37 (59%) of the matched metastases and 13/21 (62%) of the unmatched brain metastases (*P *= 0.019). In agreement, phosphorylated HER3 confirmed more frequent activation in the brain metastases, with positivity in 14/37 (37%) of the primary tumors, 24/37 (64%) of the matched metastases and 18/21 (85%) of the unmatched brain metastases (*P *= 0.046) (see Additional file 2, Table S1 and Figure S1).

Immunohistochemistry for GRB2, HIF1-alfa and phosphorylated ERK1/2, JNK1/2, ERK5 and p38 also demonstrated increased activation in the metastases compared to the primary tumors; (see Additional file 2, Table S1 and Figure S1). In contrast, phosphorylated AKT was equally high in both the primaries and metastases (see Additional file 2, Table S1). Interestingly, the non-breast derived brain metastases showed similarly high activation of the MAPK pathway together with over-expression (3+ stain) of EGFR (in 9/11 (81%) metastases (a prostate and one colon carcinoma did not) but in the absence of HER3 activation (0/11) (see Additional file 2, Table S1).

### Somatic mutation analysis

**Figure 3:**
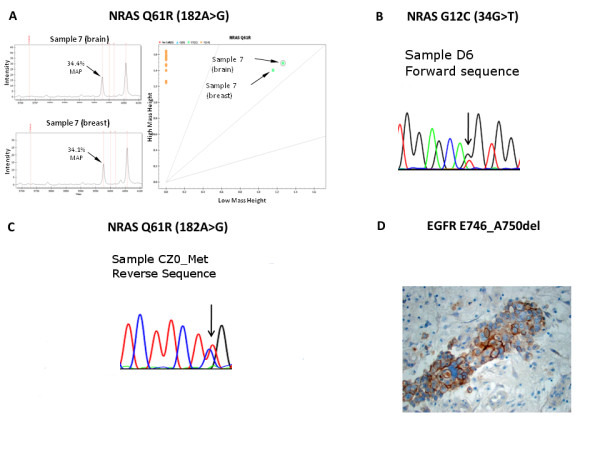
**Oncocarta and validation mutation analyses**. **A)**. Example of *NRAS *Q61R mutation identified by OncoCarta in matched pair sample #7 showing representative spectra and cluster plot. **B) **Sequence validation of *NRAS *G12C, sample D6 - lung metastasis. **C) **Sequence validation of *NRAS *Q61R, breast and brain metastasis from sample #2. **D) **Immunohistochemistry of brain metastasis (sample D4) with antibody specific to *EGFR *E746_A750del showing staining in the tumor but not the surrounding brain tissue.

OncoCarta analysis identified mutations in the brain metastases from primary breast cancers (non-autopsy cases) in *NRAS *(2/12 - 17%), and *PIK3CA *(2/12 - 17%) (Table 1 and Figure 3). Mutations were also identified in brain metastases from non-breast primaries in *EGFR *(3/9 - 33%; two lung and one kidney), *HRAS *(1/9 - 11%; lung), *KRAS *(2/9 - 22%; one colon and one lung), *NRAS *(3/9 - 33%; two lung and one kidney) and *PIK3CA *(2/9 - 22%; one melanoma and one lung).

Mutant Allele Proportions (MAPs) ranged from 9% to 80%. All these mutations were validated by immunohistochemistry (using a specific antibody raised against the protein with the *EGFR *E746_A750del mutation) or sequencing except for one each in *EGFR, HRAS *(validated by iPLEX), *NRAS *and *PIC3CA *(*validated *by HRM), where the estimated mutant allele proportion was less than 15%, and two in *PIK3CA *in which there was insufficient good quality DNA remaining to obtain sequence data. *EGFR *G719 S appeared to be found frequently by OncoCarta but could not be detected by iPLEX, using independent PCR and extension primers. The OncoCarta false-positive result appeared to be due to hairpin formation of the extension primer that occurred frequently when archival DNA was used as a template, and the yield was low.

Except for one *EGFR *mutation (Case #13; Table 1), the same somatic mutations were observed in the brain metastases with similar MAPs as in the matched primary breast tumors. It was noteworthy that the four matched pairs harboring somatic mutation in *NRAS *or *PIK3CA *also overexpressed a member of the HER family. For example, matched pair #2 had a mutation in *NRAS *and showed over-expression of HER3, matched pair #7 had a mutation in *NRAS *and showed over-expression of HER1, matched pair #9 had a mutation in *PIK3CA *and amplification of HER2 and matched pair #10 had a mutation in *PIK3CA *and overexpression of HER1 (Table 1).

Among the autopsy samples of cases with primary breast cancer, we found mutations in *EGFR *in one liver and one lymph node metastases, and a mutation in *PIK3CA *in all the samples from one case, and in a liver metastasis from another (see Additional file 2, Table S2). One *EGFR *and one *PIK3CA *mutation could be verified by sequencing or immunohistochemistry but lack of good quality DNA, and additional mutation-specific antibodies, prohibited validation of the others. All the samples from one case had the same mutation at similar MAPs (*PIK3CA *H1074R in Patient #2).

We identified *HRAS *and *PIK3CA *mutations in the basal breast cancer cell lines SUM 159 and BT20. The mutations with MAPs >25% have been reported before [19,20]: *HRAS *G12 D (MAP 53.2% in SUM159) and *PIK3CA *H1047L (MAP 50.0% in SUM159) and P539R (MAP 43.8% in BT20) but we also identified *HRAS *Q61K at MAP 24.6% in SUM159 and *HRAS *Q61K at MAP 14.1%, and *PIK3CA *H1047R at MAP 44.4% in BT20. In addition, we were also able to show that all of the mutations with MAPs >25% were present in mammospheres derived from these cell lines.

## Discussion

**Figure 4:**
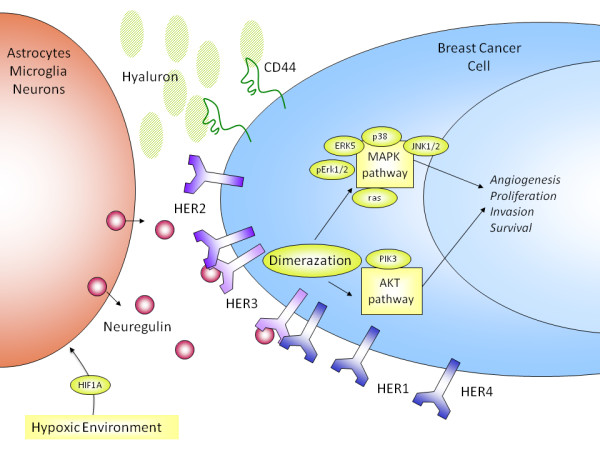
**Hypothetical mechanism of breast cancer cell colonization of the brain parenchyma**. Hypoxic conditions, (HIF1a) can mediate the elease of neuregulin 1 from neuronal cells. Neuregulin 1 is the ligand for HER3 and on binding activates the heterodimerisation of HER3-HER2, HER3-HER4 and/or HER3-HER1, leading to downstream activation of the MAPK and AKT pathways. MAPK/AKT pathways activation is related to survival, invasion, proliferation and angiogenesis. A second mechanism of colonization may relate to the enriched expression of CD44 breast cancer cells in the brain. The brain microenvironment is rich in hyaluron, the ligand for CD44, and so upon activation a series of responses maybe triggered, including cell motility.

We have collected a unique set of clinical material through collaborations with multiple institutions around the world and involving brain metastases which are rarely excised. The analysis of this resource has led to the development of hypotheses regarding the mechanisms of breast cancer colonization of the brain (Figure 4). The set of tumor samples is enriched for triple negative/basal breast cancers which is consistent with the findings of an increased propensity for basal breast cancers to metastasize to the brain [3,9,36]. An association between CD44+/CD24- frequency and a basal tumor phenotype has already been reported [37] and interestingly we observed an increased frequency of CD44+/CD24- cells in the brain metastases compared to their matched primaries. CD44+/CD24- cells have been reported to have stem cell properties and increased *in vivo *tumorigenicity [38] and the increased frequency seen in brain metastases may support this. Alternatively, this may reflect selection as a result of a high content of hyaluron, the ligand for CD44, within the brain microenvironment [39,40]. Hence, this could be an important factor in breast cancer colonization of the brain and therefore a potential axis for future therapeutic intervention [41].

In this study, brain metastases of breast cancer expressed all members of the HER family of tyrosine kinase receptors. HER2 was amplified and overexpressed in 20% of brain metastases, EGFR was overexpressed in 21% of brain metastases, HER3 was overexpressed in 60% of brain metastases and HER4 was overexpressed in 22% of brain metastases and generally mutually exclusive (Table 1). Interestingly, HER3 expression was increased in breast cancer cells residing in the brain. Neuregulin 1, the ligand for this receptor, is abundantly expressed in the brain [34,35] and is released by a variety of mechanisms including the presence of hypoxia [42]. Consistent with this, we observed the increased expression of *HIF-1alfa *in the brain metastases, likely to reflect the local hypoxic environment [43]. Increased activation of both HER3 and downstream molecules (GRB2, ERK5, ERK1/2, JNK1/2, p38) was also observed in the brain metastases. These findings prompted us to hypothesize that neuregulin/HER3 activation is an important mechanism for breast cancer cell colonization of the brain (Figure 4). As a further support to this hypothesis, increased HER3 expression has also been reported in brain metastases of lung cancer [44].

We investigated whether this association was generic to all brain metastases and found activation of the MAPK pathway in all 11 non-breast metastases to the brain. Whilst HER3 was not activated in these tumors, 9/11 tumors showed over-expression of EGFR. It has recently been shown, using animal models, that EGFR ligands mediate breast cancer metastasis to the brain and that this was abrogated by the use of EGFR inhibitor cetuximab [14]. The combination of lapatinib and trastuzumab has been shown to have a synergistic, antiproliferative effect against ErbB2-positive breast cancer cells *in vitro *[45]. It is possible, therefore, that a combination of anti-HER therapies could be effective in the treatment of both breast and non-breast metastases to the brain.

In order to activate downstream signaling pathways, HER3 requires heterodimerization with other members of the HER family following binding by neuregulin [46] and even basal levels of the other HER proteins may be sufficient to participate in the activation of these pathways. Hence, combination therapy against the HER family, even in the absence of over-expression or amplification of HER2, may be of clinical benefit for a larger proportion of breast cancer patients such as those with HER2 negative disease. Recently, a study showed benefits for a small group of HER2-negative patients in the phase III National Surgical Adjuvant Breast and Bowel Project (NSABP) B-31 trial that were HER2 negative by FISH and had less than 3+ staining intensity by HercepTest^® ^(Dako, Carpinteria, CA, USA) [47]. Furthermore, another study suggested that the spectrum of patients who may benefit from trastuzumab-based therapies could be expanded to include patients with metastatic breast cancer without HER-2 amplification but who express transmembrane neuregulin, the ligand of HER3 [48]. It has also been reported in non-HER2 over-expressing xenograft models of prostate and breast cancer that pertuzumab, an inhibitor of HER3/HER2 heterodimerization, can inhibit tumor growth [49].

For the first time, we have identified somatic mutations in genes related to the AKT/MAPK signaling pathways, such as *EGFR*, *PIK3CA*, *KRAS*, *HRAS *and *NRAS*, in brain metastases of breast cancer and other types of cancer. In addition, we have analyzed multiple autopsy samples from six cases that had a primary breast cancer, and found additional *EGFR *and *PIK3CA *mutations in breast cancers that metastasized to various sites including the brain. Thus, simply targeting the HER family of receptors may not be sufficient for complete treatment response. This analysis highlights additional *actionable *targets [50] that may prove effective for the treatment of some brain metastasis, such as PI3 kinase inhibitors.

Taken together, these findings are striking and show another facet of the cell evolution landscape [51], highlighting the possibility of cancer cells resisting targeted treatment to molecules such as HER2 or EGFR by acquiring oncogenic mutations in downstream pathways. This has been shown *in vitro *with activating PIK3CA mutation [23] and herein we demonstrate an *in vivo *example of this possible scenario using human tumors. In another clinical angle, patients currently treated with the anti-EGFR monoclonal antibodies cetuximab and panitumumab can also acquire resistance to this therapy due to downstream mutations in the *ras *gene [24]. Interestingly, animal models have suggested that downstream NF-kappaB inhibitory drugs may play a role in the treatment of patients with defined mutations in *KRAS *[52].

Interestingly the Mutant Allele Proportion (MAP) was sometimes as low as 10%. Such low proportion mutations, which would often be missed by direct sequencing could reflect the presence of stromal (or brain) contamination in the samples, tumor heterogeneity and amplification or deletion of the mutant or wild type alleles. However, the fact that the same MAP was often observed in both the primary and the brain metastasis, and in the multiple samples from an autopsy case, might suggest that these metastases were not seeded by a single cell but by groups of cells from the primary tumor. This has also been shown by next generation sequencing, whereby the mutant allele frequency for some mutations was similar between a basal-like primary breast cancer and its matched brain metastasis [53]. However, it is also evident that significant genomic evolution occurs during metastasis, since most mutations identified in this metastasis, and one from a primary lobular breast cancer, were more prevalent in the metastasis than in the respective primary tumours [53,54]

## Conclusions

In conclusion, we provide evidence to support a role of HER3 and other HER family receptors in the ability of cancer cells to colonize the brain. The data are intriguing and support the possibility that tumors with low expression of HER2 may respond to trastuzumab, lapitinib or combinations of HER family receptor inhibitors since even basal levels may enhance the signaling through homo/hetero-dimerization of the other receptors. However, caution should be exercised because of the possible presence of downstream oncogenic mutations that may drive treatment resistance. These therapeutic modalities may therefore add another dimension to the treatment of triple negative and basal-like cancers where currently, no targeted therapy is available.

## Abbreviations

CISH: chromogenic *in situ *hybridization; DASL: cDNA-mediated Annealing, Selection, extension, and Ligation; EGFR: epidermal growth factor receptor; ER: estrogen receptor; FFPE: formalin fixed-paraffin embedded; GEO: Gene Expression Omnibus; HER: human epidermal growth factor receptor; HRM: High Resolution Melt; IDC: invasive ductal carcinoma; MAPs: Mutant Allele Proportions; NSABP: National Surgical Adjuvant Breast and Bowel Project; NST: non-specific type; PgR: progesterone receptors.

## Competing interests

Leonard Da Silva and Sunil Lakhani hold an USA registered patent relating to the data in this manuscript. All the other authors declare no conflict of interest.

## Authors' contributions

LDS analysed the immunohistochemical markers, accrued and collated the data, carried out statistical and gene expression analysis and drafted the manuscript. PK and ACV analysed immunohistochemical markers, and accrued and collated the data. NW, CES and PTS supervised gene expression analyses and drafted the manuscript. EP, PF, AS, MF, RB, MB and MC identified patients with brain metastases in their institutions, collected samples and performed initial tumor classification. LR, SP, PK and AL performed immunohistochemistry and participated in the construction of TMAs. KK, NK, BJM and BR participated in the study design. SB, SH and JB performed mutation analyses. HD, AD and SF performed validation of EGFR mutations. GCT and SRL conceived the study, supervised the experiments and drafted the manuscript.

## Acknowledgements

Leonard Da Silva and Ana Cristina Vargas are recipients of PhD Fellowships from the Ludwig Institute of Cancer Research. Leonard Da Silva is enrolled with the *"Universidade Federal de São Paulo, Escola Paulista de Medicina, Curso de Pós-Graduação, Doutorado, Departamento de Anatomia Patológica, São Paulo, Brazil"*. Peter Simpson is a recipient of a fellowship from the National Breast Cancer Foundation. Georgia Chenevix-Trench and KumKum Khanna are Senior Principal Research Fellows of the NHMRC. RLB is a Cancer Institute NSW Fellow. We also acknowledge the help of staff within anatomical pathology, RBWH, Brisbane, the animal house facility at UQ AIBN, Brisbane, Casey Wright from the Thoracic Research Laboratory, School of Medicine, at the UQ, and Clay Winterford and his staff from the UQ/QIMR Histotechnology facility, and Macky Edmundson in the sequencing facility at QIMR. We would like to thank Sequenom Inc. for providing the primer sequences used for HRM, and, in particular, we thank Darryl Irwin for his help.

## Supplementary Material

Additional file 1 **Supplementary methodologies**. This file contains information of how the morphological review and TMA creation were performed. It also contains information on protocols for immunohistochemistry and chromogenic *in situ *hybridization, RNA extraction and Real-Time RT-PCR, DASL gene expression profiling, cell line analysis and culture, oncoCarta somatic mutation analysis protocols, high resolution melt analysis and iPLEX genotyping protocols. Click here for file

Additional file 2 **Supplementary results**. This file contains tables and figures regarding all immunohistochemistry data, extra gene expression and mutation results, and HER family gene expression by RT-PCR. Click here for file
