# Supplementary material for: HER3 and downstream pathways are involved in colonization of brain metastases from breast cancer
Source: Breast Cancer Res. 2010 Jul 6;12(4):R46. doi: 10.1186/bcr2603 (PMC2949633; doi:10.1186/bcr2603)
Supplement: Additional file 1 — Supplementary methodologies. This file contains information of how the morphological review and TMA creation were performed. It also contains information on protocols for immunohistochemistry and chromogenic in situ hybridization, RNA extraction and Real-Time RT-PCR, DASL gene expression profiling, cell line analysis and culture, oncoCarta somatic mutation analysis protocols, high resolution melt analysis and iPLEX genotyping protocols. [file bcr2603-S1.doc]

**Additional file 1**

**Morphological review & TMA creation**

Haematoxylin & eosin (H&E) sections were reviewed by three pathologists (LDS, MC, SRL) to confirm the diagnosis, assess morphology and grade the tumours according to the World Health Organization criteria[55]. Tissue microarrays (TMA) were built using the tissue arrayer, model MTAI (Beecher Instruments, Inc, Sun Prairie, WI 53590 USA) to facilitate screening of clinical samples using immunohistochemistry and in situ hybridization. H&E slides were dotted by LDS in order to take representative 0.6mm diameter cores of each tumour for analysis. Two cores were taken from each tumour. Two sets of TMAs were built. The first TMA set comprised 29 primary breast cancers and their matched brain metastases and 22 unmatched brain metastases. The second TMA set comprised 10 primary breast cancers and their matched brain metastases, as well as the 11 non-breast brain metastases. Slides and paraffin blocks from 38 tumor samples (primary breast cancer and metastases to multiple sites, including brain) from 8 autopsy cases of patients who died of metastatic breast cancer were also available.

**Immunohistochemistry and chromogenic *in situ* hybridization**

Four micron thick sections of the paraffin blocks were cut on to silane-coated slides. Immunohistochemistry was performed using the Envision® dual link system (Dakocytomation, Denmark) according to the manufacturer’s recommendations. Supplementary table 1 summarises all antibody details. Antigenic retrieval for all antibodies (except smooth muscle actin (SMA) which did not require any antigen retrieval and EGFR – 5 minutes chymotrypsin digestion) required two minutes pressure cooking (105^o^C) in EDTA (pH8.0) buffer. Positive and negative controls were included in all runs and all slides were analysed by at least two pathologists (LDS, PP or SRL) using double headed optical light microscope. HER2 staining and scoring used a minimum 30% percent cut-off of positivity with strong complete membrane staining to regard a case as HER2 overexpressed (3+). The same criteria were used for EGFR, HER3 and HER4 and assessed in the periphery of the tumors. For all other antibodies, samples were considered positive if more than 10% of tumor cells were stained in one or both cores on the TMA. Cellular localization (membrane, cytoplasm, nuclear), staining intensity (negative, weak/1+, moderate/2+, strong/3+) and percentage/number of positively stained neoplastic cells was recorded. A tumor was regarded as ‘basal’ if any of the following markers were positive (CK5/6, CK14,CK17, p63, SMA, EGFR). The EGFR, HER3, HER4, CD44 and CD24 were assessed on whole sections and not as double stain. Digital slide images are available at <http://aperio.qimr.edu.au/>. The username is LeoExternal and password leoext1 (case sensitive) and the images are under the head “List all digital images”. Chromogenic *in situ* hybridization was performed following instructions of the Zymed Spot-Light®HER2 CISH^TM^ Kit (Zymed, California, USA). Briefly, the paraffin embedded sections were dewaxed and subjected to heat and enzyme digestion treatments followed by denaturation and hybridization to labelled nucleic acid probes. Immunodetection was performed and 3, 3’-Diaminobenzidine tetrachloride was used for visualization. Slides were counterstained with haematoxylin. A positive control for HER2 amplification was included in each run. Signals were counted under a brightfield microscope and results were classified into diploid, polysomy, low amplification and high amplification according to the manufacture’s recommendation. Immunohistochemistry using an antibody raised against the E746_A750del mutated EGFR protein was performed to validate Oncocarta® data. Myoepithelial cells of normal breast tissue, which are normally positive for wild-type EGFR, were also used as negative control for the E746_A750del mutation antibody.

**Supplementary Table 1 – Details of Antibodies Used in Immunohistochemistry**

| Antibody | Company | Clone – Dilution |
| --- | --- | --- |
| ER | Novocastra | 6F11 – 1:100 |
| PR | Novocastra | 1A6 – 1:500 |
| HER2 | DAKO | Herceptest® |
| HER3 | Novus Biologicals | RTJ2 - 1:80 |
| HER4 | Santa Cruz | C-18 - 1:100 |
| EGFR | Zymed | 31G7 - 1:100 |
| p63 | DAKO | 4A4 – 1:400 |
| SMA | DAKO | 1A4 – 1:100 |
| p53 | DAKO | D07- 1:100 |
| CD44 | DAKO | DF1485 – 1:100 |
| CD24 | Serotec | SN3 – 1:200 |
| CK5/6 | DAKO | D5/16B4 – 1:50 |
| CK14 | Neomarkers | LL002 – 1:50 |
| E-cadherin | Zymed | HECD-1 1:20 |
| CK19 | DAKO | RCK108 – 1:25 |
| CK7 | DAKO | OV-TL12/30 – 1:500 |
| CK17 | DAKO | E3 -1:100 |
| CK8/18 | Novocastra | 5D3- 1:100 |
| Phospho HER3 | Cell Signalling | Tyr^1289^(21D3) -1:300 |
| Phospho AKT | Invitrogen | pT^308^-1:300 |
| Phospho ERK1/ERK2 | Invitrogen | pTpY ^185/187^-1:300 |
| Phospho JNK1/2 | Invitrogen | pTpY ^183/185^-1:300 |
| Phospho ERK5 | Invitrogen | pTpY ^180/182^-1:300 |
| p38 | Invitrogen | pTpY ^218/220^-1:300 |
| Mutated-EGFR | Cell Signalling | E746-A750del(6b6) – 1:100 |
| Ki-67 | Dako | MIB1 -1:100 |
| GRB2 | Cell Signalling | 1:100 |
| HIF1-alfa | Cell Signalling | 1:100 |

**RNA extraction and Real-Time RT-PCR**

Three 5μm thick FFPE sections of clinical samples were cut and de-waxed in two lots of xylene and two lots of 100% ethanol, 10 minutes each. RNA was extracted using the High Pure RNA Paraffin Kit® (Roche Applied Science, Mannheim, Germany) according to the manufacturer’s protocol. RNA was quantified using the NanoDrop® ND-1000 (NanoDrop Technologies, DE, USA). Relative expression levels of *HER3, HER2, EGFR, HER4, HIF1-alfa and CCNH* was assessed using RT-PCR. Complementary DNA (cDNA) was synthesized from 240ng of total RNA using random hexamers and the SuperScript^TM^ III Reverse Transcriptase kit (Invitrogen, Carsbald, CA, USA). TaqMan One-Step UniversalMaster Mix (Applied Biosystems) was used for all reactions. TaqMan reaction was done in a standard 96-well plate format with ABI 7500 OneStepPlus PCR system. For data analysis, raw deltaCt (dCt) was first normalized to an endogenous control gene (RPL13a) for each sample to generate normalized dCt. The normalized dCt was then calibrated to a cell line pool reference (MCF-7, SKBR-3 and MDA-MB-231) to generate a ddCt. In the final step of data analysis, the ddCt was converted to fold change (2^-ddCt^) relative to the reference allowing comparison between samples.

**DASL gene expression profiling**

Gene expression profiling was performed using the DASL assay (cDNA-mediated annealing, selection extension and ligation, Illumina Inc., California, USA) to interrogate the DASL Cancer Panel that contained 512 cancer related genes [56-58]. All protocols were as specified by Illumina Inc. Briefly, RNA (250ng) was converted to cDNA through a reverse transcription reaction with biotinylated oligo-d(T)_18_ and random nonamers. Gene specific oligonucleotides (three unique pairs for each of the 512 genes) were annealed to the biotinylated cDNA, the duplexes were bound to streptavidin conjugated paramagnetic particles to remove non-hybridized oligos. The annealed oligos were then extended and ligated (to incorporate an address sequence and primer site) to generate amplifiable products. These products were subjected to PCR amplification using fluorescently labeled (Cy3 and Cy5) primers. The labeled PCR products were hybridized to a Sentrix array or Beadchip. Following hybridization, the arrays were scanned with a BeadArray Reader (Illumina) and data was extracted using BeadStudio version 3 software (Illumina). Samples exhibiting a median hybridization intensity across all probes of <600 (background corrected) were excluded from analysis. Individual probes with a BeadStudio detection score greater than 0.99 in more than 15 conditions were included which left 1234 probes in the analysis. Array data transformation and normalization (per chip normalized to 50th percentile and per gene normalized to median) was done in Genespring® version 7.0 software (Agilent Technologies, Santa Clara, USA). Hierarchical clustering was performed using Pearson Correlation and Average Linkage clustering algorithm. Principal component analysis was also performed. A linear model was fitted (limma) and a moderated t-statistic was performed for differential expression. DASL data is available from Gene Expression Omnibus (<http://www.ncbi.nlm.nih.gov/geo/>, Accession number GSE14690).

**Cell lines**

SUM159 and BT20 mammospheres were grown in 5 ml of serum-free DMEM/F12 (NSA) medium containing freshly added 20 ng/ml rhEGF (R&D Systems), 10 ng/ml rhFGF (R&D Systems), 4 ug/ml heparin, 10% proliferation supplement (NeuroCult®, Stem Cell Technologies Inc.), 2% BSA (Sigma). Breast cancer cell lines MCF-7, MDA-MB-231 and SKBR-3 were cultured in Dulbecco’s Modified Eagle’s Medium (DMEM) plus 10% heat-inactivated fetal bovine serum (FBS), 2 mmol/L glutamine, and 1% penicillin G-streptomycin solution.

**OncoCarta somatic mutation analysis**

Oncogenic mutations in tumour samples were profiled using the OncoCarta Assay Panel v1.0, which offers rapid, parallel analysis of 238 mutations across 19 common oncogenes. All reactions were performed according to the OncoCarta v1.0 User’s Guide (Sequenom, San Diego, CA). PCR amplification was carried out using 20 ng DNA as a template for each Assay. Post-PCR treatment by shrimp alkaline phosphatase was followed by the TypePLEX Extend Reaction. Following this step, CLEAN Resin (Sequenom) was added to the mixture to remove extraneous salts that could interfere with the MALDI-TOF analysis. Allelotyping was determined by robotically spotting 15 nl of each extension product onto a SpectroCHIP II, which was subsequently read by the MassARRAY Compact Analyzer. Some mutations were validated with a second OncoCarta analysis, using just a subset of primers that detect the relevant *EGFR* mutations. Poor quality samples were identified by evaluating the primer extension rates. All samples in which more than 10% of assays had less than 50% primer extension were defined as ‘poor quality’ and removed from further analysis.

**High Resolution Melt Analysis**

For cases with sufficient DNA available, we carried out High Resolution Melt (HRM) analysis to validate mutations in *KRAS, HRAS, NRAS* and *PIK3CA* identified with the OncoCarta Assay. Primers used for HRM Analysis were the same as those used in the OncoCarta Assay Panel and are shown in Supplementary Table 2. PCR reactions for HRM for *KRAS, HRAS, NRAS* and *PIK3CA* were performed on a LightCycler 480 (Roche Diagnostics, Mannheim, Germany) in 10ul final volume containing 1x LightCycler 480 High Resolution Melting Master Mix (Roche Diagnostics), 500 nM forward primer, 500 nM reverse primer, 30 mM MgCl_2_ and 10 ng genomic DNA. The cycling conditions were as follows: Pre-incubation at 95°C for 5min, followed by 26 cycles of 95°C for 10 s, touchdown from 65°C to 53°C (-0.5°C/cycle) for 10 s and 72°C for 10 s, followed by a further 19 cycles at 53°C annealing temperature. The melting program consisted of one cycle at 95°C for 1 min, 40°C for 1 min and then continuous fluorescent reading from 65 to 95°C at 25 acquisitions per °C. HRM data were analysed using the LightCycler 480 Gene Scanning Software (Roche Diagnostics) as previously described [59].

**Supplementary Table 2 - Primers used for HRM Analysis**

| **Primer name** | **Targeted**  **Mutation** | **Primer Sequence** | **Product Size** |
| --- | --- | --- | --- |
| NRAS_6 - 1st Primer | Q61R | ACGTTGGATGTCGCCTGTCCTCATGTATTG | 99 |
| NRAS_6 - 2nd Primer |  | ACGTTGGATGCCTGTTTGTTGGACATACTG |  |
| PIK3CA_6 - 1st Primer | E545K | ACGTTGGATGTACACGAGATCCTCTCTCTG | 90 |
| PIK3CA_6 - 2nd Primer |  | ACGTTGGATGTAGCACTTACCTGTGACTCC |  |

**Sequencing**

For cases with sufficient DNA available, and an OncoCarta Assay result of > 30% mutant allele proportion (MAP) for *KRAS, HRAS, NRAS* and *PIK3CA* we used direct sequencing for validation. Primers for sequencing (Supplementary Table 3) were designed using the web-based programme Primer 3 (<http://frodo.wi.mit.edu/primer3/>). PCR reactions were performed in a final volume of 20 μl and contained 15 ng DNA, 200 nM of each primer, 250 μM dNTPs, 1× PCR buffer with 2 mM MgCl_2_, and 1 U i-STAR Taq polymerase (Scientifix, Clayton, Australia). Touchdown amplification was as follows : 94^o^C for 12 min, followed by four sets of four cycles of 94^o^C for 30 s, 61^o^C to 55^o^C for 45 s and 72^o^C for 30 s, with the annealing temperature dropping 2^o^C after each set of four cycles, followed by 30 cycles of 94^o^C for 30 s, 55^o^C for 45 s and 72^o^C for 30 s, and a final extension of 72^o^C for 7 min. PCR reactions were purified with the QIAGEN PCR purification kit and sequenced using Big-Dye (version 3.1) sequencing chemistry and the PE Applied Biosystems 377 sequencer. The resulting chromatograms were compared with wild type samples which were sequenced as controls.

**Supplementary Table 3 - Primers for sequencing**

| **Primer name** | **Targeted**  **Mutation** | **Primer Sequence** | **Product Size** | |
| --- | --- | --- | --- | --- |
| KRAS1-2/4_(G12C/G13D)-F | G12C and G13D | TTAACCTTATGTGTGACATGTTCTAA | 171 |  |
| KRAS1-2/4_(G12C/G13D)-R |  | TGGATCATATTCGTCCACAAAA |  |  |
| NRAS2_(G12C)-F | G12C | GATGTGGCTCGCCAATTAAC | 175 |  |
| NRAS2_(G12C)-R |  | CTCACCTCTATGGTGGGATCA |  |  |
| NRAS6_(Q61R)-F | Q61R | CACCCCCAGGATTCTTACAG | 173 |  |
| NRAS6_(Q61R)-R |  | TCCGCAAATGACTTGCTATT |  |  |
| PIK3CA9_(H1047L)-F | H1047L | TGAGCAAGAGGCTTTGGAGT | 190 |  |
| PIK3CA9_(H1047L)-R |  | GGTCTTTGCCTGCTGAGAGT |  |  |

**iPLEX genotpying**

For cases with sufficient DNA available, we used Sequenom’s MassARRAY system and iPLEX technology to validate mutations in *NRAS, PIK3CA* and *EGFR* identified with the OncoCarta Assay.  The design of oligonucleotides was carried out according to the guidelines of Sequenom Inc. and performed using MassARRAY Assay Design software (version 1.0).  Four-Plex PCR amplification of amplicons containing variants of interest was performed using Qiagen HotStart Taq Polymerase on a Perkin Elmer GeneAmp 2400 thermal cycler with 5 ng genomic DNA in a 2.5 μl reaction.  Primer extension reactions were carried out according to manufacturer’s instructions for iPLEX chemistry.  Assay data were analysed using Sequenom TYPER software (Version 3.4).

**Supplementary Table 4 - Primers for iPLEX**

| **Primer name** | **Mutation** | **Primer Sequence** | **Product Size** |
| --- | --- | --- | --- |
| NRAS_Q61R_q1 | Q61R | ACGTTGGATGCCTGTTTGTTGGACATACTG |  |
| NRAS_Q61R_q2 |  | ACGTTGGATGTCGCCTGTCCTCATGTATTG |  |
| NRAS_Q61R_q3 |  | TGGCACTGTACTCTTCT |  |
| PIK3CA_E545K_q1 | E545K | ACGTTGGATGTACACGAGATCCTCTCTCTG |  |
| PIK3CA_E545K_q2 |  | ACGTTGGATGTAGCACTTACCTGTGACTCC |  |
| PIK3CA_E545K_q3 |  | AGAAAATCTTTCTCCTGCT |  |
| PIK3CA_H1047R_iPlex-F | H1047R | ACGTTGGATGAACTGAGCAAGAGGCTTTGG |  |
| PIK3CA_H1047R_iPlex-R |  | ACGTTGGATGTCCATTTTTGTTGTCCAGCC |  |
| PIK3CA_H1047R_iPlex-Ext |  | ATGAAACAAATGAATGATGCAC |  |
| HRAS_G13S_iPlex-F | G13S | ACGTTGGATGAATGGTTCTGGATCAGCTGG |  |
| HRAS_G13S_iPlex-R |  | ACGTTGGATGGACGGAATATAAGCTGGTGG |  |
| HRAS_G13S_iPlex-Ext |  | CGCACTCTTGCCCACAC |  |
| NRAS_G12C_iPlex-F | G12C | ACGTTGGATGAGTGGTTCTGGATTAGCTGG |  |
| NRAS_G12C_iPlex-R |  | ACGTTGGATGGACTGAGTACAAACTGGTGG |  |
| NRAS_G12C_iPlex-Ext |  | GCTTTTCCCAACACCAC |  |
| EGFR_E746_A750del_iPlex-F | E746_A750del | ACGTTGGATGGATCCCAGAAGGTGAGAAAG |  |
| EGFR_E746_A750del_iPlex-R |  | ACGTTGGATGTCGAGGATTTCCTTGTTGGC |  |
| EGFR_E746_A750del_iPlex-Ext |  | AATTCCCGTCGCTATCAA |  |
| PIK3CA_R38H_iPlex-F | R38H | ACGTTGGATGGGGGTATTTTCTTGCTTCTT |  |
| PIK3CA_R38H_iPlex-R |  | ACGTTGGATGCCAAATGGAATGATAGTGAC |  |
| PIK3CA_R38H_iPlex-Ext |  | ATGGTTATTAATGTAGCCTCA |  |
