# Supplementary material for: HER3 and downstream pathways are involved in colonization of brain metastases from breast cancer
Source: Breast Cancer Res. 2010 Jul 6;12(4):R46. doi: 10.1186/bcr2603 (PMC2949633; doi:10.1186/bcr2603)

**Additional file 2**

**Table 1 –** Immunohistochemistry results

| **Antibody** | **Primary breast**  **tumor**  **N/T (%)** | **Matched brain**  **metastases**  **N/T (%)** | **Unmatched**  **brain metastases**  **N/T (%)** | **Non-breast**  **brain metastases**  **N/T (%)** |
| --- | --- | --- | --- | --- |
| **ER** | 15/37(40%) | 15/37 (40%) | 4/21 (23%) | NP |
| **PR** | 9/37 (24%) | 9/37 (24%) | 5/21 (19%) | NP |
| **HER2** | 7/34 (20%) | 7/35 (19%) | 5/24 (20%) | NP |
| **Triple negative** | 19/29(56%) | 20/35(48%) | 10/22(45%) | NP |
| **CK5/6** | 9/26 (34%) | 9/26 (34%) | 2/16 (12%) | NP |
| **CK14** | 15/35 (42%) | 16/36 (44%) | 8/16 (50%) | NP |
| **CK17** | 5/26 (19%) | 5/26 (19%) | 5/16 (30%) | NP |
| **EGFR** | 6/26 (23%) | 6/26 (23%) | 3/16 (18%) | 9/11 (81%) |
| **p63** | 0/26 | 0/26 | 1/15 (5%) | NP |
| **SMA** | 6/26 (23%) | 6/26 (23%) | 2/15 (13%) | NP |
| **Basal-like** | 20/37 (54%) | 21/35 (60%) | 11/22 (50%) | NP |
| **CD44** | 5/20 (25%) | 13/20 (65%) | 10/22 (45%) | NP |
| **CD24** | 5/22 (22%) | 2/22 (9%) | 3/22 (14%) | NP |
| **CD44^+^CD24^-^** | 5/20(25%) | 11/20 (55%) | 10/22(45%) | NP |
| **p53** | 16/26 (61%) | 19/26 (73%) | 14/20 (70%) | NP |
| **KI-67** | 19/37(51%) | 32/37 (86%) | 18/21 (85%) | NP |
| **CK19** | 24/26 (92%) | 24/26 (92%) | 15/15 (100%) | NP |
| **CK8/18** | 14/26 (53%) | 14/26 (53%) | 15/15 (100%) | NP |
| **E-cadherin** | 19/26 (73%) | 19/23 (82%) | 18/18 (100%) | NP |
| **HER3** | 11/37 (29.7%) | 22/37 (59%) | 13/21 (62%) | NP |
| **HER4** | 7/35(20%) | 6/26 (23%) | 5/24 (20%) | NP |
| **Phospho HER3** | 14/37 (38%) | 24/37 (64%) | 18/21 (85%) | 0/11 |
| **Phospho AKT** | 32/37 (86%) | 32/37 (86%) | 15/21 (71%) | 11/11 (100%) |
| **Phospho ERK1/2** | 28/37 (75%) | 36/37 (97%) | 20/21(95%) | 11/11 (100%) |
| **Phospho JNK1/2** | 26/37 (70%) | 34/37 (91%) | 19/21 (90%) | 11/11 (100%) |
| **Phospho ERK5** | 29/37 (78%) | 36/37 (97%) | 20/21 (95%) | 11/11 (100%) |
| **p38** | 29/37(78%) | 37/37 (100%) | 21/21 (100%) | 11/11(100%) |
| **GRB2** | 13/36 (36%) | 16/36 (44%) | 11/22 (50%) | NP |
| **HIF1-alfa** | 9/37 (24%) | 20/35(48%) | 8/16 (50%) | NP |

**Legend:** N = number of tumor cases showing positivity; T = total number of cases assessable for the antibody specified; % = percentage of cases showing positivity; NP = not performed; A tumor was regarded as ‘basal’ if any of the following markers were positive (CK5/6, CK14, CK17, p63, SMA, EGFR,) in more than 10% of cells. Triple negative tumors were negative for ER, PR and HER2. CD44+/CD24- immunohistochemistry was assessed on serial sections and positivity was expression in >10% cells. X^2^ test with Yates correction (95% confidence interval) showed significant differences between matched primaries and metastases as follow: phospho-Her3 p =0.046, phosphor-ERK1/2 p= 0.017, phosphor-ERK5 p=0.032, phosphor-JNK p= 0.037, p38 p=0.008, HER3 p=0.019, HIF1-alfa p=0.009 and CD44 p=0.026. GRB2 showed a trend p=0.1

**Figure 1 -** Scatter plots showing distribution of positivity for ER, phospho-HER3, phospho-ERK1/2 and phospho-ERK5 across matched and unmatched samples. Panel on left shows the correlation of immunohistochemical score between primary tumor (x-axis) and the matched metastasis (y-axis). The panel on the right shows the immunohistochemical score (y-axis) for each marker in individual unmatched metastases (x-axis).

**
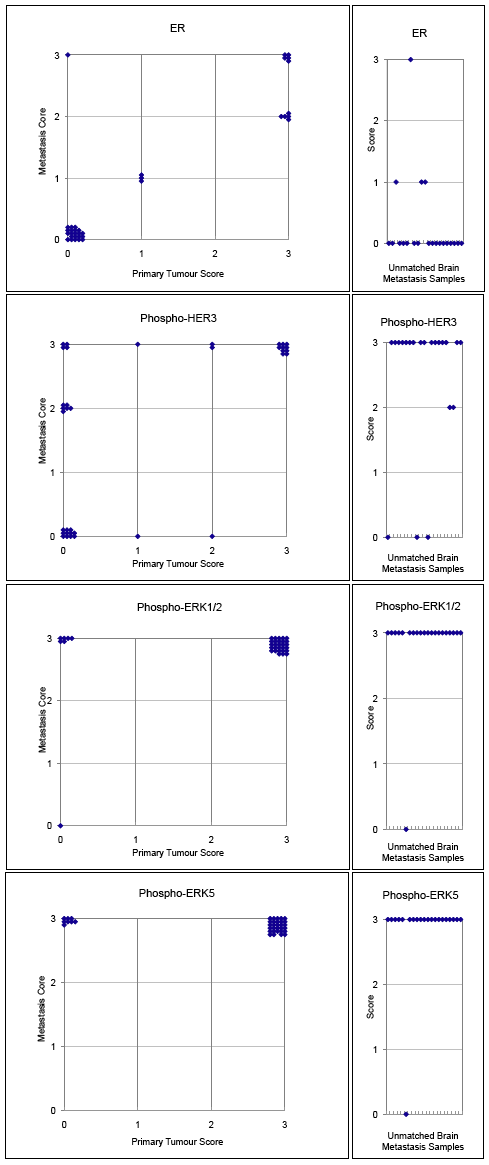
**

**Figure 2:** Principal component analysis using the 27 gene list from figure 2B showing good separation between brain metastases (red squares) and primaries (blue squares).

**
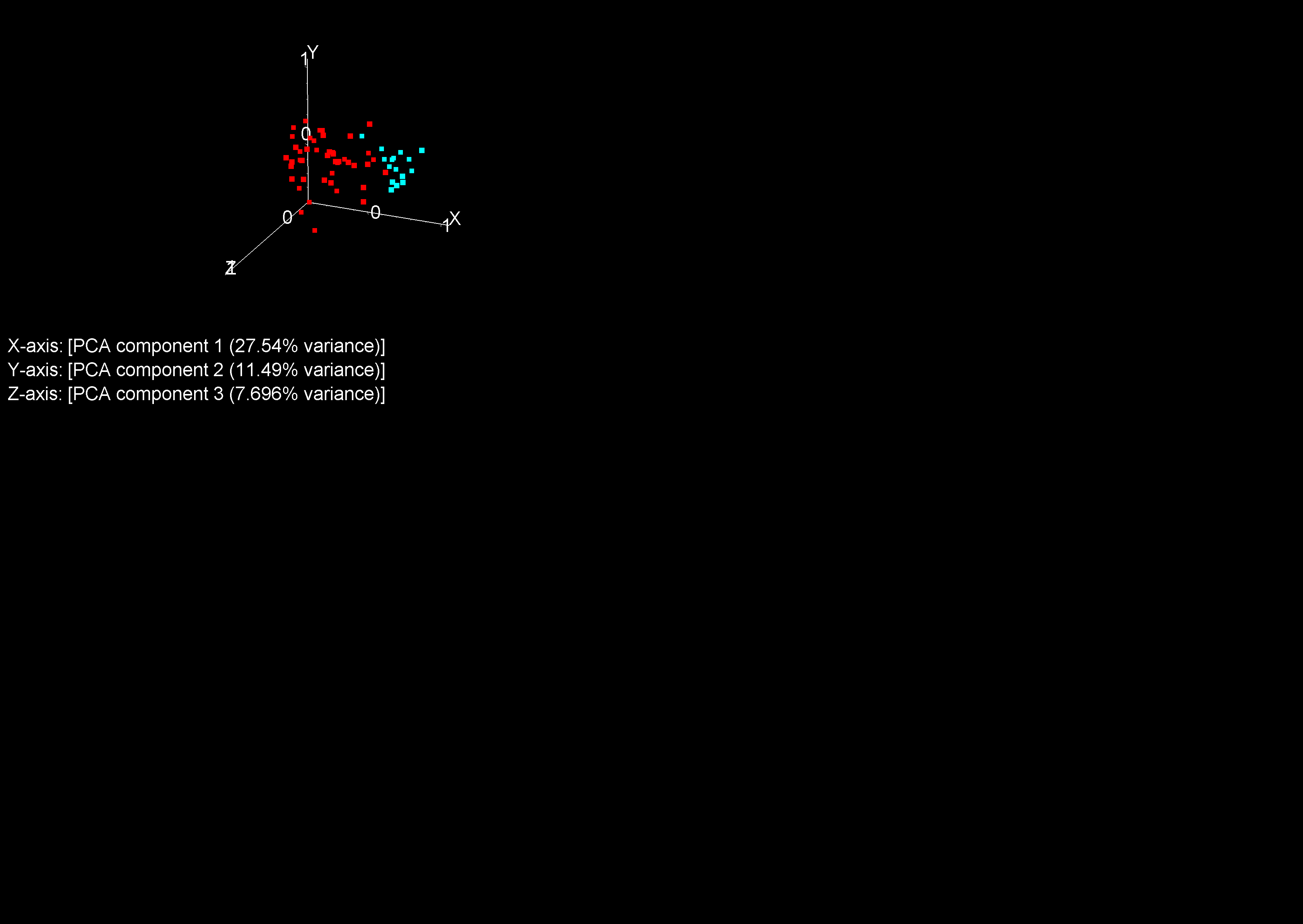

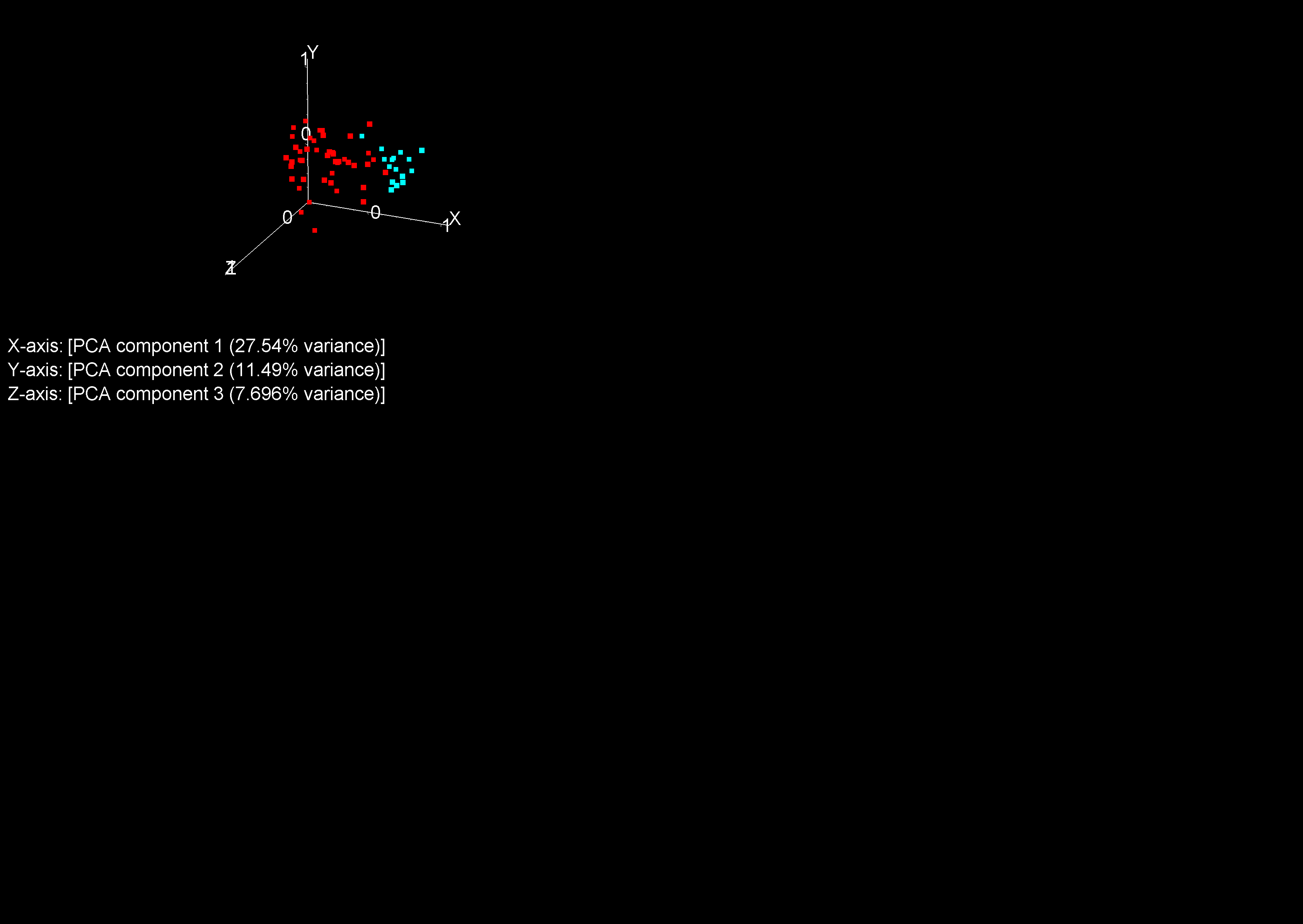
**

**Table 2:** Somatic mutations in autopsy samples

| **Case ID#** | **Site** | **GRADE** | ***EGFR*** | | | ***PIK3CA*** | | |
| --- | --- | --- | --- | --- | --- | --- | --- | --- |
|  |  |  | **Mutation** | | **MAP** | **Mutation** | | **MAP** |
| **1** | **Breast** | **3** |  | |  |  | |  |
|  | **Brain** |  |  | |  |  | |  |
|  | **Lung** |  |  | |  |  | |  |
|  | **Mediastinal lymph node** |  |  | |  |  | |  |
|  | **Adrenal gland** |  |  | |  |  | |  |
| **2** | **Brain** |  |  | |  | H1047R^I, S^ | | 57.6% |
|  | **Liver** |  |  | |  | H1047R^I,Y^ | | 54.9% |
|  | **Breast** | **3** |  | |  | H1047R^I,Y^ | | 54.7% |
|  | **Peritoneum** |  |  | |  | H1047R^I,Y^ | | 65.0% |
|  | **Lung** |  |  | |  | H1047R^I,Y^ | | 53.1% |
| **5** | **Axillary lymph node** |  |  | |  |  | |  |
|  | **Breast** | **3** |  | |  |  | |  |
|  | **Adrenal gland** |  |  | |  |  | |  |
|  | **Lung** |  |  | |  |  | |  |
| **6** | **Ovary** |  |  | |  |  | |  |
|  | **Brain** |  |  | |  |  | |  |
|  | **Brain** |  |  | |  |  | |  |
|  | **Breast** | **2** |  | |  |  | |  |
| **7** | **Liver** |  | E746_A750del^O, I,A^ | | 12.7% | R38H^X, Y^ | | 20.7% |
|  | **Brain** |  |  | |  |  | |  |
|  | **Axillary lymph node** |  |  | |  |  | |  |
| **8** | **Lymph node** |  | H773_V774insNPH^NVP^ | | 27.4% |  | |  |
|  | **Pituitary/hypothalamus** |  |  | |  |  | |  |
|  | **Pleura** |  |  | |  |  | |  |
|  | **Breast** |  |  | |  |  | |  |
|  | **Pancreas** |  |  | |  |  | |  |
|  |  |  |  | |  |  | |  |
| MAP = Mutant Allele Proportion estimated by OncoCarta | | | | |  |  | |  |
| NVP = no validation possible because no DNA remained | | | | |  |  | |  |
| O = validated by repeat OncoCarta analysis using a subset of assays that included primers for EGFR G719S, D770_N771insG, and E746_A750del mutations  S = validated by sequencing  A = validated by immunohistochemistry using a mutation-specific antibody | | | | | | | | |
| I = validated by iPLEX using diferent primers from the OncoCarta assay | | | | | |  |  |  |
| X = iPLEX didn't work for this sample | | |  |  | |  |  |  |
| Y = Sequencing did not work for this sample | | | |  | |  |  |  |

**Figure 3**: RT-PCR and DASL assay for HER receptors genes and HIF1-alfa summary


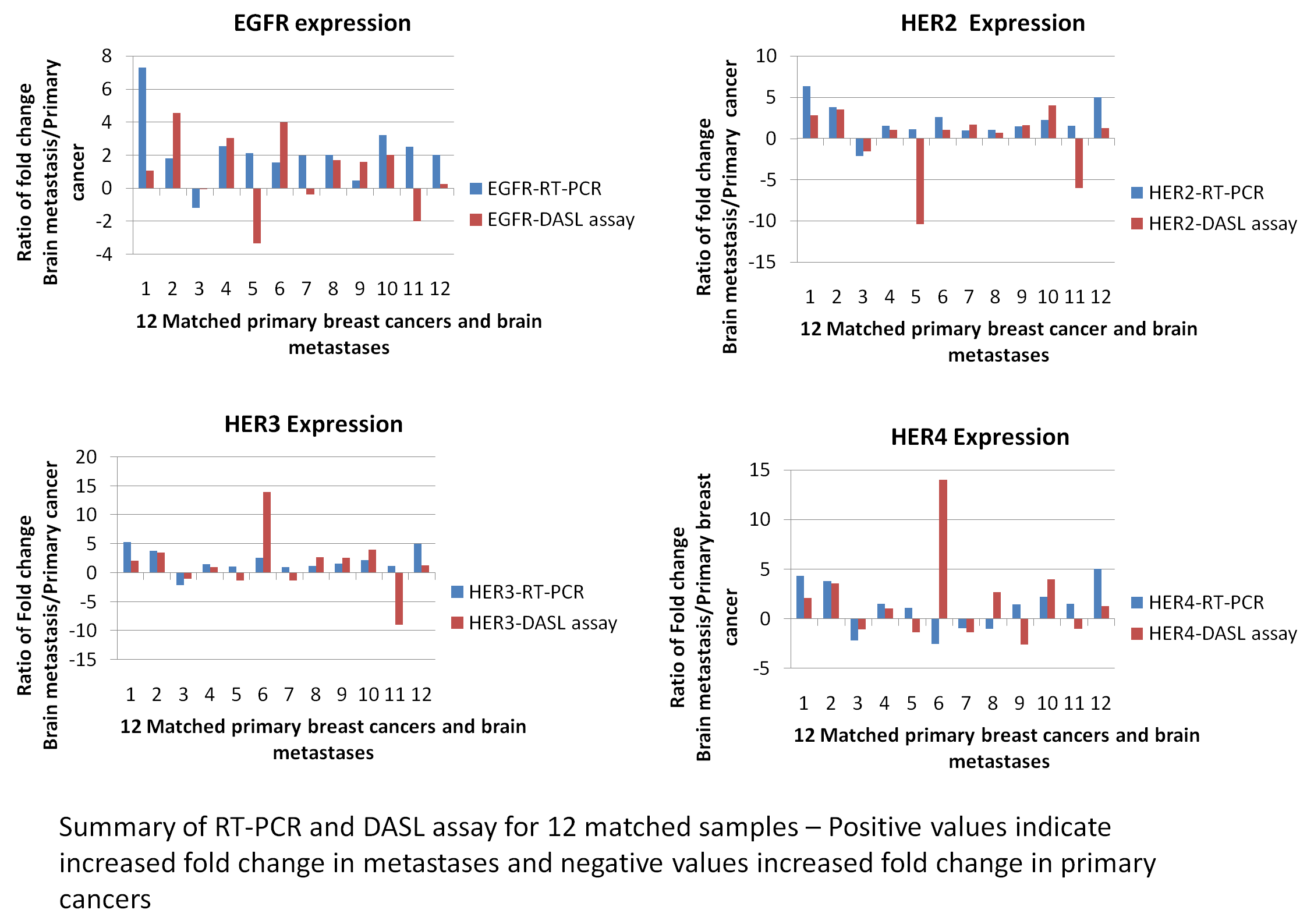


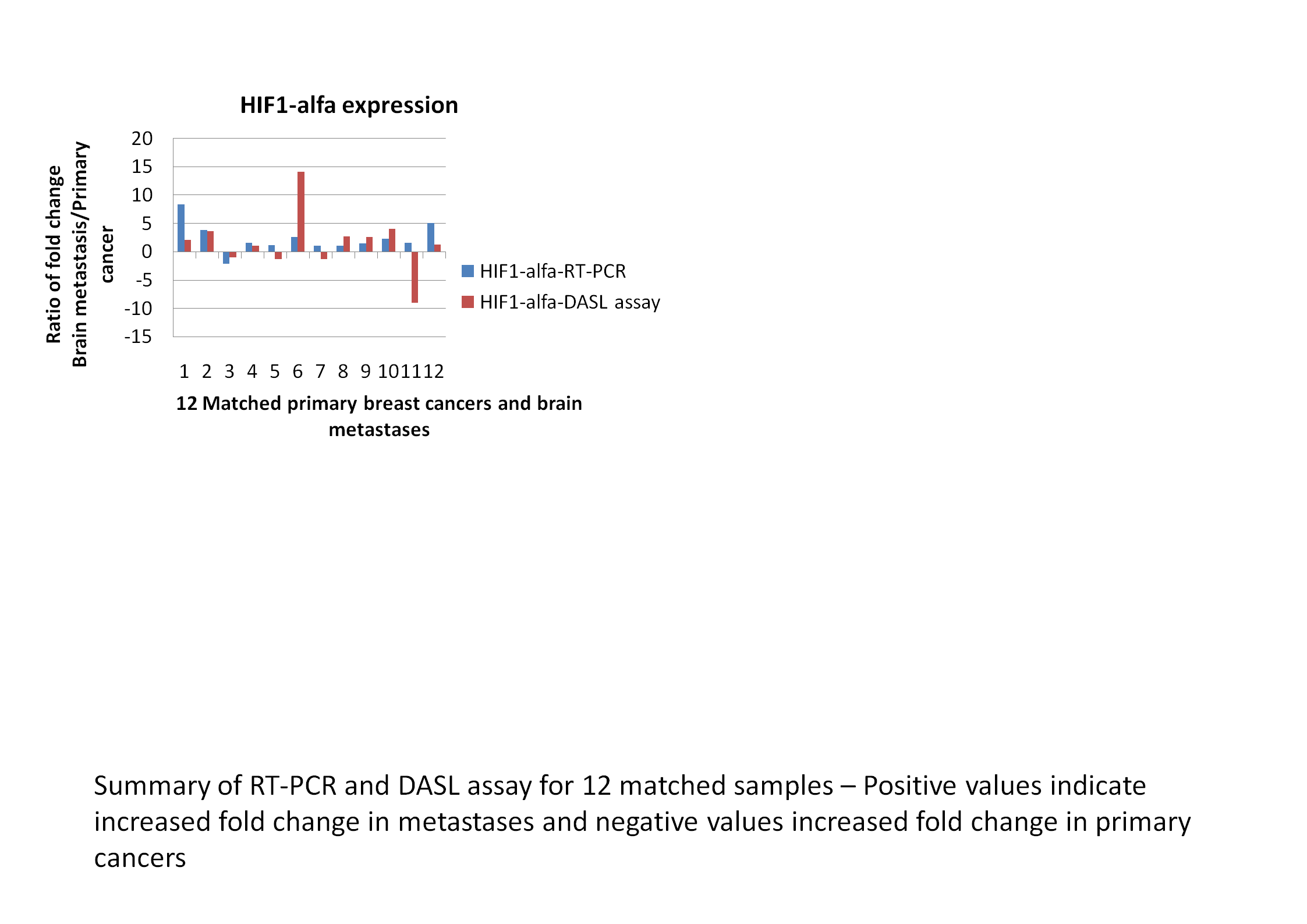

Supplement: Additional file 2 — Supplementary results. This file contains tables and figures regarding all immunohistochemistry data, extra gene expression and mutation results, and HER family gene expression by RT-PCR. [file bcr2603-S2.doc]
